# Mental Strain as a Disease

**Published:** 1870-10

**Authors:** 


### EDITORIAL DEPARTMENT.

---

*Mental Strain as a Disease.*—Dr. B. W. Richardson, of London, has recently referred to the importance of studying the influence of impressions made through the mind upon the organs of the body.

He contends that when the mental powers are so greatly overtasked, as they are at present times, this study claims the immediate attention of the general as well as the psychological physician. Dr. Richardson in describing the different classes of men who are engaged in labors which call forth the energies of the mind, divides them into "copyists, authors, artists, speculators, professionals and students. He defines the nature of the work of these respectively, and the diseases to which they are subject as the effect of their labors.

Turning to the diseases in detail, Dr. Richardson places not only paralysis, insanity, epilepsy, intermittent action of the heart, and arterial enervation with symptoms simulating aneurism, but also diabetes and cancer, as diseases directly connected with mental strain in persons who have an hereditary tendency to them.

As relates to insanity itself, the author holds an opinion different from that commonly entertained, in his asserting that work, and even hard work of a mental character, does not of itself bring on the disease, but that by such work the organs of the body which are purely physical are most severely injured. Insanity is, he thinks, a disease growing out of ignorance and mental inactivity; so that, in fact, the sluggish parts of the population are the most determinate producers of insanity, and the educated and most intelligent parts of our population are the producers and propagators of the more serious and fatal of organic diseases.

But in reasoning on the share which undue strain on the brain —the material organ of the mind—has in causing insanity, there is one important distinction to be constantly remembered, viz., that between the faculties purely intellectual on the one side, and the feeling, emotions and passions, which make up the largest portion of the functions of the brain, on the other. More commonly it will be found that insanity is the result of excessive indulgence and contentions in these latter, rather than in a simply severe taxing of the intellect alone.

This belief will be fully borne out by the history of genius in all its varied manifestations. And again, the intellect of a student is often supposed to have become weakened, if not lost, by too close and continuous labor, when, in fact, the result was chiefly due to irregular hours, strong drinks, and not seldom tobacco or opium."

